# Improving Agility by Managing Shared Libraries in Microservices

**DOI:** 10.1007/978-3-030-58858-8_20

**Published:** 2020-08-18

**Authors:** Saulo S. de Toledo, Antonio Martini, Dag I. K. Sjøberg

**Affiliations:** 6 IT University of Copenhagen, Copenhagen, Denmark; 7 University of British Columbia, Vancouver, BC Canada; University of Oslo, Oslo, Norway

**Keywords:** Cross-company study, Multiple-case study, Software quality, Qualitative analysis, Architectural technical debt

## Abstract

Using microservices is a way of supporting an agile architecture. However, if the microservices development is not properly managed, the teams’ development velocity may be affected, reducing agility and increasing architectural technical debt. This paper investigates how to manage the use of shared libraries in microservices to improve agility during development. We interviewed practitioners from four large international companies involved in microservices projects to identify problems when using shared libraries. Our results show that the participating companies had issues with shared libraries as follows: coupling among teams, delays on fixes due to overhead on libraries development teams, and need to maintain many versions of the libraries. Our results highlight that the use of shared libraries may hinder agility on microservices. Thus, their use should be restricted to situations where shared libraries cannot be replaced by a microservice and the costs of replicating the code on each service is very high.

## Introduction

A microservices architecture may be considered a kind of agile architecture. Over the years, large companies such as Amazon and Netflix shared their success histories with microservices on dozens of presentations^1^, always highlighting how such architectural style helped them to be agile and surpass many of the limitations and impediments they had in their previous monolithic software solutions. Since then, many other companies and practitioners tried to learn about microservices and adopted them in their projects.

However, systems that use microservices may become more complex than monolith systems [8]. Practitioners are still struggling with the adoption of this architectural style in their projects, and there is not much knowledge about Architectural Technical Debt (ATD) in microservices [10].

ATD is a metaphor used to describe architectural suboptimal decisions that, in exchange of benefits in the short term, incurs future additional costs for the software. There are many studies on ATD in general but few on ATD in microservices and no discussion about agility. Our previous study [10] investigates what is ATD in microservices through a qualitative case study in a single company, Lenarduzzi and Taibi [5] presents a position paper about code debt on microservices, also in a case study in a single company, Bogner et al. [3] performed a qualitative case study in 10 companies to explore evolvability assurance processes for microservice-based systems. The three studies have distinct scopes.

In this study, we investigate the practice of using shared libraries in companies that use microservices, and how do these companies manage such libraries in order to improve agility. We define a *shared library* as a piece of software developed in-house containing a collection of resources used by several components. Externally developed components such as frameworks and language support extensions are not considered shared libraries in this study. Shared libraries are used as a black box by the different components, have their own version management and are copied and bundled together with the components.

Taibi and Lenarduzzi [9] have shown that the use of shared libraries may be a microservice bad smell and have proposed solutions for removing the smell. We extend that work by presenting an expanded list of issues and solutions, and do it in the context of different companies.

We pose the research questions as follows:

- **RQ1:** Which practical issues when using shared libraries in microservices hinder agility in organizations?
- **RQ2:** Which solutions do developers apply to solve such issues?

In order to answer these questions, we conducted a multiple-case study in four large international companies that use microservices. The remainder of this paper is structured as follows: Sect. 2 presents our background, Sect. 3 our methodology, Sect. 4 our results, Sect. 5 our discussion and threats to validity. Section 6 concludes and outlines future work.

## Background

Using microservices architecture is an approach that decomposes a single application into a collection of small and loosely coupled services; such services are autonomous, independent of each other and run on separate processes [6]. A few other characteristics are also taken in consideration while defining microservices, such as loose coupling, organization around business capabilities and ownership by small teams.

Microservices may improve agility by allowing teams to focus on small pieces of software, facilitating aspects like change, scalability and testing. As it raises new ways of developing software, it also raises new kinds of ATD [10]. If properly managed, the accumulation of ATD may be beneficial to the software development, but it is necessary to know when the debt should be avoided and how to prevent its accumulation [7].

ATD is based on financial terms and has three main concepts [2]: *debt*, which describes a sub-optimal solution that yields short-term benefits, but recurring to the later payment of some interest; *interest*, which is the additional cost that has to be paid because of the accumulation of debt; and *principal*, which is the cost of refactoring in order to remove the debt.

## Methodology

We conducted a multiple-case study in four large international companies, with more than 1000 employees. For confidentiality reasons, the companies are named *A*, *B*, *C* and *D*, respectively. The studied projects operate in the domains as follows, respectively: financial systems, healthcare systems, city management and transport mobility.

We interviewed six architects: one from Company A, two from each of Companies B and C, and one from Company D. We conducted semi-structured interviews that lasted from 30 min to one and a half hours. We discussed several aspects of architecture beyond the scope of this investigation, such as architectural issues and solutions while using microservices. The questions in the interview guide relevant to this study are available at https://bit.ly/ImprAgilitySL. Three of the interviews were conducted face-to-face. The three other ones were conducted through remote audio calls due to the physical distance between the parts.

## Results

### The Issues Caused by Using Shared Libraries

**Fig. 1.**
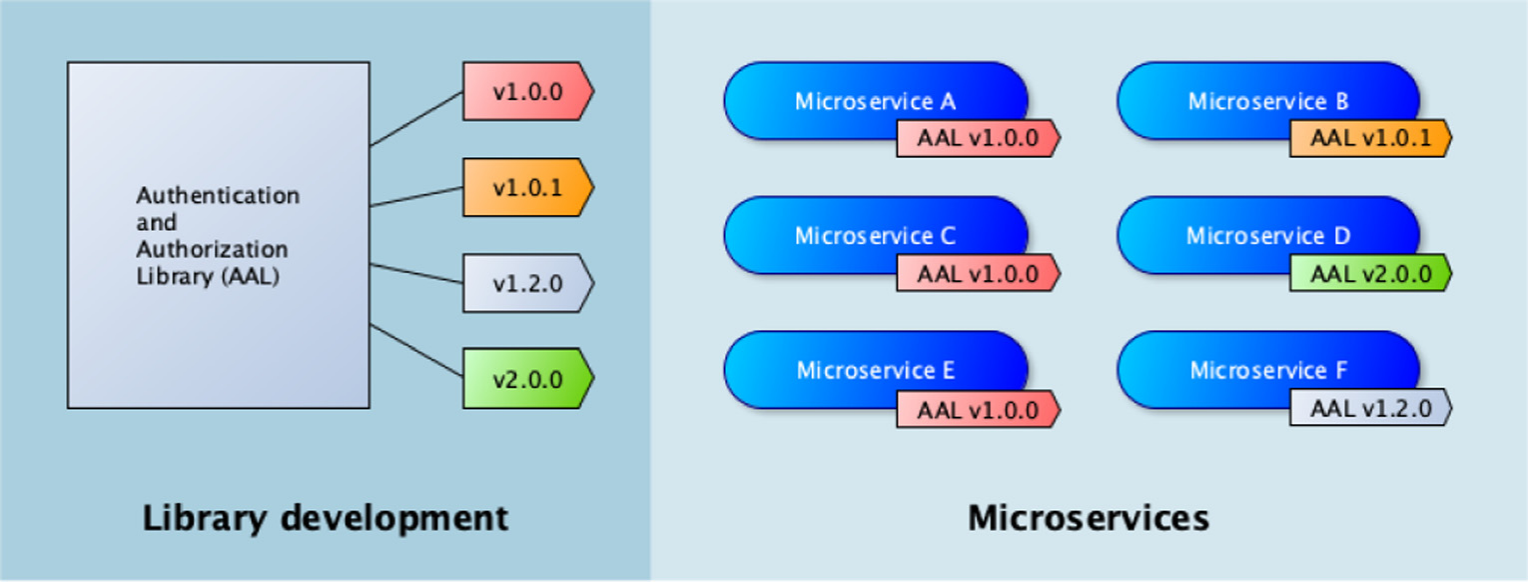
Shared libraries example

Table 1 shows which issues related to shared libraries were found in which companies. We refer to the those issues by using their IDs between parenthesis in the following paragraphs. The context related to the issues discussed below is illustrated in Fig. 1, an example reported by Company B: A team is assigned to create and maintain a library for authentication and authorization. Versions of the library are regularly released with fixes or new functionalities. Other teams are assigned to develop microservices. Eventually and due to several reasons, several microservices end up using distinct versions of the library. We present below the causes and implications of such circumstances for each company in the context of the projects we investigated.

**Table 1.** Issues reported by companies as the result of using shared libraries

ID	Issue	Company			
		A	B	C	D
1	Impossibility to update library in service due to priorities	X		X	
2	Need to maintain too many versions of the library	X		X	
3	Impossibility to update library in service due to breaking changes			X	
4	Delays while waiting for fixes		X	X	X
5	Early adopters refusing to migrate	X			
6	Failures due to unknown use cases		X	X	
7	Failures after library upgrades		X	X	
8	Overhead to library maintainers		X	X	X
9	Dependent agile teams	X	X	X	X

Company A could not migrate all the clients to a newer version of a library right after its release. Distinct teams have different priorities: some services are critical, some are secondary, some have more urgent updates (1). Such a scenario required libraries maintainers to be active in supporting previous versions of their libraries that were still being used in production (2). Even in situations where the library was supposed to be updated soon, the company experienced delays in the process due to other priorities (1). In addition, the company also identified situations where early adopters resisted to migrate (5), since a new version of the library was released right after they finished the integration of the previous version in their project.

In Company B, the developers experienced a number of system breaks. Later they identified that part of the breaks were caused by the use of libraries in many unforeseen and untested situations (6). In addition, Company B also noticed an overhead on library maintainers (8) and consequent delays. Since the functionality was provided by the libraries, the teams using them had to wait for the fixes, which caused delays in new microservices releases (4). In some situations, the new versions of the libraries caused new issues that prevented the microservices to be released in production right away (7).

Company C, similarly to Company A, found itself in a situation where it was not possible to migrate all the clients, which required teams to support many deprecated versions of libraries (2). Breaking changes and internal roadmap priorities were some of the factors that prevented developers to use new versions of the libraries (3 and 1). The use of shared libraries became a bottleneck, causing failures on microservices (6 and 7), delays while waiting for fixes (4) and an unexpected amount of extra work for library developers (8).

Company D reported delays in delivering new functionalities as the most damaging issue connected to the use of shared libraries (4). The library developers had to handle an extensive amount of change requests, including requests for additional features and fixes (8). The microservices developers were frequently blocked while waiting for the arrival of the new versions of the libraries.

In all four companies, there was a clear dependency (coupling) among the microservices developers and the library teams (9).

### How to Manage Issues Regarding the Use of Shared Libraries

All the companies reported that the use of shared libraries should be reduced as much as possible. Company B reported that many libraries implemented trivial functionality that could be implemented by the microservices themselves, and the fixes could be implemented by the teams, reducing the delays caused by third-party developers. Company D suggested that well-defined and well-documented interfaces of their own implementations were important for guiding practitioners when they did not use shared libraries to provide required functionality.

**Fig. 2.**
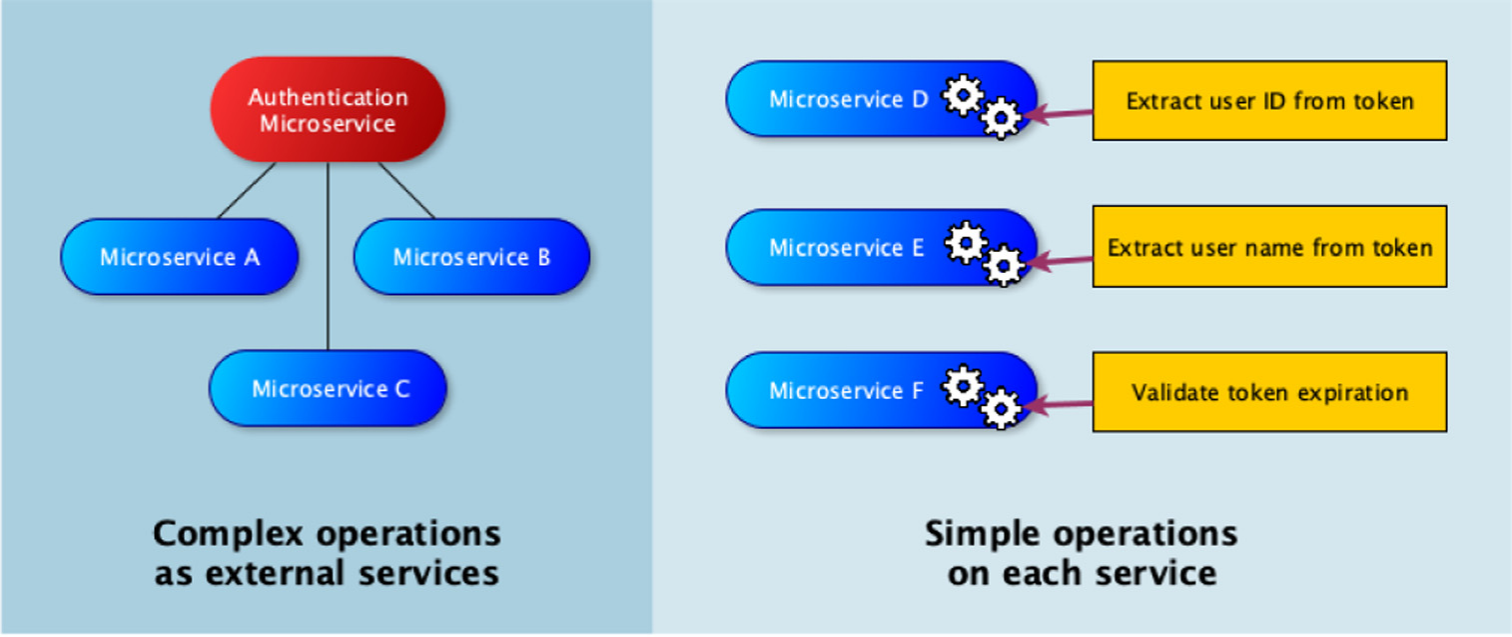
How to handle shared functionality

Figure 2 shows solutions proposed by the companies for the issues caused by the use of shared libraries. Considering the example presented in Fig. 1, simple functionalities, such as extracting an ID or user name from a token, could be implemented by the services themselves. Such a functionality is easy to implement, usually by using a well-known technique that can be learned by the developers, and that does not require the use of an entire library. On the other hand, some functionalities are complex and could involve, as in our example, many security steps. In such circumstances, an external microservice with a well-defined interface, good documentation and a versioning policy should be maintained by a separate team. Well-defined interfaces should not be changed unless in exceptional cases, meaning that internal bug fixes may be conducted without the other services noticing it, and new functionality may be added without breaking previous behavior unless a breaking change is strictly necessary. Such a scenario reduces the need for changes in the other microservices that are using the aforementioned interfaces. Finally, if there are important reasons for not using one of the approaches above, the use of shared libraries may be acceptable. Similar approaches may be found in other migration reports. Balalaie et al. [1], for example, moved common libraries to microservices when they migrated to such an architecture style. Hasselbring et al. [4] argue that code should not be shared among microservices because teams and applications should be as independent and loosely coupled as possible.

## Discussion and Threats to Validity

Our results suggest that using shared libraries in some contexts impacts on the development flow, causing delays, reducing development velocity and hindering agility. In such cases, shared libraries are an ATD that may lead to costly interest if not managed properly. By sharing the experience from other practitioners on issues and solutions, we can prevent others from having to pay high software maintenance costs later.

We answer the research questions introduced in Sect. 1 by listing the issues (RQ1) raised by the use of shared libraries and by presenting corresponding solutions (RQ2). The issues we identified do not seem connected to any specific application domain; the practitioners from the different companies complained about similar issues and solutions. We do not claim that shared libraries should never be used. However, their use should be controlled to prevent high costs. There are also drawbacks of such an approach. For example, it may incur additional latency; performance may decrease due to network as opposed to in-memory invocations; reliability may decrease since the service might not be reachable; and complex functionality may not be possible to be implemented in a distributed system. Such drawbacks should be carefully considered in practical situations.

Companies should also consider the reasons for replacing their shared libraries. There may be alternative solutions, such as improving processes for development, testing and quality assurance, which should be considered when the drawbacks of moving to services may be more costly than using shared libraries.

Regarding the validity of this study, we consider the following threats: (i) The interviewees may have interpreted the concept of shared libraries differently. We mitigated this threat by asking the interviewees to clarify if they were talking about libraries developed internally or about external dependencies; (ii) Our sample of interviewees was small from each company, we do not know how representative the opinions in this study were for the investigated companies. Still, the sample was heterogeneous and the practitioners were located in three different countries, with projects from four different companies; (iii) There might be factors that the interviewees were not aware of or did not express in the interviews, such as the quality of the implementations and management issues.

## Conclusions and Future Work

In four Europe-based companies, we identified a set of issues that reduce development velocity and hinder team agility while using shared libraries in microservices. We highlighted two solutions: creating additional microservices or implementing the code in the microservices themselves. Although these solutions have been reported by Taibi and Lenarduzzi [9], we went beyond their work by presenting and discussing a more comprehensive list of issues, and relating them all to the different companies. Our results suggest that the use of shared libraries may increase the complexity of the system, which in turn decreases development agility, cause delays and raises maintainability costs. Our results do not indicate that shared libraries should not be used at all, but if there are no acceptable alternatives, they should be used rather carefully as they often generate costly interest. As an alternative to the use of shared libraries, simple functionalities should be implemented by each microservice, whereas complex functionalities should be implemented by external microservices with well defined interfaces, good documentation and adequate versioning policies.

As future work, we propose a further investigation of the problem, increasing the size of the sample and looking for practitioners with different experiences. As part of this investigation, we propose to look for a decision process supported by the factors that influence the trade-off between using a shared library and a microservice. We would also like to investigate the problem and their solutions with other architectural styles, like Service Oriented Architecture, in order to identify whether there are other solutions proposed by practitioners that could be used in microservices. In addition, we would like to investigate the external dependencies and how moving to them could affect our results.

## Footnotes

1 Examples of presentations are “Mastering Chaos” by Josh Evans (Netflix, 2016), “Amazon and the Lean Cloud” by Werner Vogels (Amazon, 2011) and “What We Got Wrong: Lessons from the Birth of Microservices” by Ben Sigelma (Google, 2018).
